# Age-related changes in hand function – it’s not just about muscle strength

**DOI:** 10.3389/fnhum.2026.1779077

**Published:** 2026-03-20

**Authors:** Rachel N. Logue Cook, Susan H. Brown

**Affiliations:** School of Kinesiology, University of Michigan, Ann Arbor, MI, United States

**Keywords:** aging, dexterity, hand, intervention, tactile acuity

## Abstract

The hand is a remarkable organ that is essential for daily living, functional independence, and quality of life. Age-related declines in sensorimotor function, however, lead to impaired hand dexterity, fine force control, and coordinated movement that are independent of comorbid conditions such as arthritis or peripheral neuropathies. Despite a large body of research illustrating how these changes impact daily activities, routine clinical assessment of mobility in older adults has primarily focused on postural control or the ability to generate maximum grip strength. This mini-review provides an overview of functional hand declines associated with aging including the importance of low force control and somatosensory feedback. In addition, the benefits of targeted training programs to improve hand sensorimotor function and the impact of factors such as sex differences, physical inactivity, and occupation on hand use are described. The goal of this review is to increase awareness of the importance of maintaining or improving hand function in our increasingly aging segment of society – it’s not just about muscle strength.

## 1 Introduction –the grasping hand

The hand is a remarkable and complex organ that provides humans with a vast array of functional, creative, and expressive skills. Despite its small size – approximately 1 % of total body area (Rhodes et al., 2013) - it is comprised of 27 bones and controlled by over 30 muscles located in the palm and forearm (Informed Health.org, 2006). This anatomical arrangement allows for versatility of hand shapes needed for most activities of daily living, ranging from full hand power grasps to delicate precision pinch movements. From a force control perspective, the size of motor units comprising hand muscles is quite small compared to other limb muscles which allows for precise gradation of force. As a result, the neuromuscular system can accurately modulate hand force from levels ranging from 2 to 10 N during manipulation of fragile objects (Gorniak et al., 2010; Xu et al., 2024) to almost 900 N during maximum grip force tasks (Perna et al., 2016). As a sensory organ, the hand is exquisitely sensitive to touch and movement with over 17,000 tactile receptors providing feedback related to mechanical deformation of the skin (Johansson, 1978). Muscle and joint receptors provide additional feedback regarding muscle length and force, and joint position (Jones and Lederman, 2006). It comes as no surprise, therefore, that philosophers, writers, and scientists have referred to the hand as “the cutting edge of the mind” (Bronowski, 1973), “the visible part of the brain” (Immanuel Kant in Longo, 2025), and “the seeing hand” (Erik Moberg in Dellon, 1990; Keller, 1908). Precise control of the hand is dependent upon a complex distributed network of several movement-related areas of the cerebral cortex (Davare et al., 2011). During dexterous visually-guided movements, visual information about object characteristics and location is transmitted to specialized areas of the parietal cortex where it is transformed into intrinsic joint and muscle coordinates essential for the planning of specific hand movements (Fattori et al., 2017; Jeannerod et al., 1995). This information is relayed to premotor areas via parieto-frontal pathways where further planning refinement occurs (Filimon, 2010; Vesia et al., 2017). The final stages in the production of descending hand motor commands via the corticospinal tract relies extensively on the integration of hand-related input from premotor and somatosensory areas (Lemon et al., 1998; Tazoe and Perez, 2017).

The importance of sensorimotor cortices in the control of hand movements is reflected by the disproportionately large hand representation in these areas with approximately 20% of the motor strip dedicated to hand control (Jones and Lederman, 2006; Yip et al., 2025). Further, over 50% of the corticospinal tract terminates on motor neurons in the cervical region of the spinal cord responsible for precise control of the hand (Lemon, 2008; Roze et al., 2025). Excellent comprehensive reviews focused on the central control of hand movements include those by Jones and Lederman (2006), Sobinov and Bensmaia (2021), and Latash (2015).

**Figure 1:**
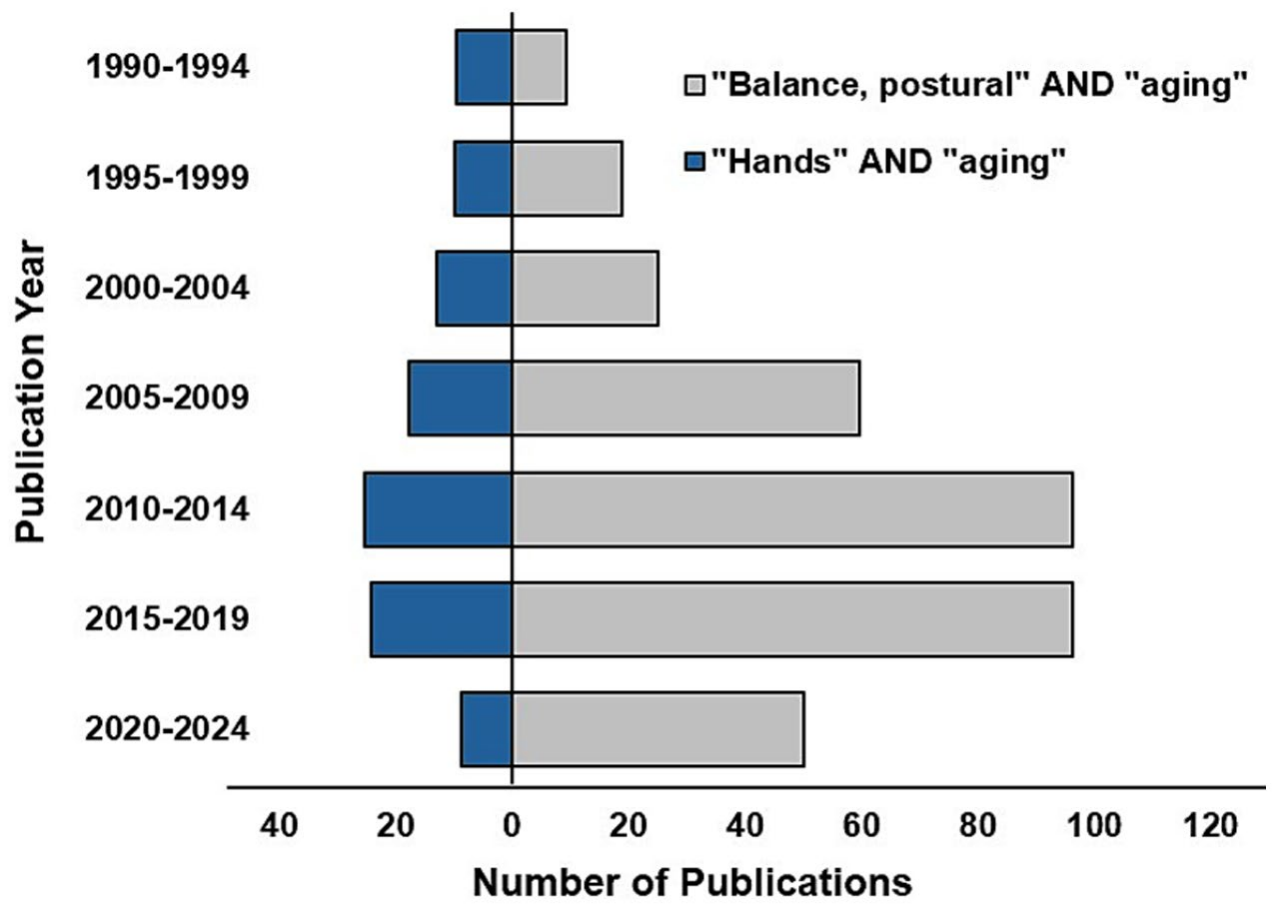
Number of publications in PubMed related to hand function (blue) compared to balance and posture (gray) between 1990 and 2024. MeSH terms included [“Hands” AND “aging”] and [“Balance, postural” AND “aging”].

From an aging and movement perspective, however, most research has focused on age-related changes in balance, associated fall risk, and loss of independence in comparison to hand function. This is illustrated in Figure 1, in which we conducted a brief analysis of peer-reviewed articles published in the biomedical database PubMed between 1990 and 2024. Of those few studies with a hand focus, a large percentage examined the basis for and consequences of declining maximum grip strength. Findings demonstrating that grip strength is a predictor of hospital admissions, the development of comorbid conditions such as diabetes and arthritis, and cardiovascular and cancer-related mortality have led to the suggestion that maximum grip strength should be considered a biomarker of health status (Bohannon, 2019). While the importance of maximum hand strength has been clearly demonstrated, clinical awareness of other aspects of declining hand function affecting, for example, dexterity and somatosensation remains poor. Thus, the purpose of this mini-review is to highlight age-related changes in hand dexterity, strength and somatosensation that can adversely impact activities of daily living and functional independence among older adults.

## 2 Age-related changes affecting hand function

Just as with other areas of the body, the hands are prone to declines in several systems that accompany the aging process. Around age 60, the number of motor units decreases (Campbell et al., 1973; Carmeli et al., 2003; Faulkner et al., 2007; Larsson et al., 2019) due to a systemic loss of muscle fibers (Faulkner et al., 2007) and denervation of these fibers from motor neurons (Larsson et al., 2019). Muscle fibers may be reinnervated by a different motor neuron, although this leads to larger motor units which are primarily comprised of slow twitch muscle fibers (Hunter, 2025; Larsson et al., 2019) with decreased motor unit firing rates (Borzuola et al., 2020; Carmeli et al., 2003; Newton et al., 1988), thus leading to difficulty controlling force (Carmeli et al., 2003; Larsson et al., 2019). Aging is also associated with changes to the structure and function of sensory receptors. The density of touch receptors decreases with age (Bruce, 1980; Carmeli et al., 2003; Decorps et al., 2014; García-Piqueras et al., 2019; McIntyre et al., 2021), particularly the Meissner corpuscles (Bruce, 1980) and Merkel’s discs (García-Piqueras et al., 2019) which are both responsible for detecting fine, discriminative touch. Muscle spindles, the primary sensory receptors involved in proprioception, also undergo age-related changes in the small muscles of the hands which can include increased capsule thickness (Swash and Fox, 1972) and a decrease in the number of intrafusal fibers (Kröger and Watkins, 2021; Liu et al., 2005; Swash and Fox, 1972).

In addition to changes in the sensory and motor systems separately, integration between the two systems is also affected by declines in peripheral nerve conduction velocity (Huang et al., 2009; Jena and Acharya, 2023; Palve and Palve, 2018; Stetson et al., 1992; Tong et al., 2004; Verdú et al., 2000), particularly in the median nerve (Huang et al., 2009; Tong et al., 2004). Age-related neural changes also occur in brain structures involved in the planning and execution of hand movements and force generation. These changes include reduced connectivity and reorganization of sensorimotor networks (Seidler et al., 2010) and neurometabolic alterations including reductions in inhibitory neurotransmitters (Cuypers et al., 2018; Levin et al., 2019). Significant neuronal loss also occurs in the cerebellum which plays an important role in the planning, monitoring, and updating of descending motor signals controlling the hand (McElroy et al., 2024). When age-related declines persist at the neurophysiological level, behavioral declines in hand function emerge. Perhaps the most well-documented aspect of the aging hand is the loss of strength, both whole hand and pinch strength. Hand grip strength peaks in the third and fourth decade of life and then decreases substantially after age 50 (Beenakker et al., 2010; Sternäng et al., 2015; Wang et al., 2018). Across the lifespan, grip strength will decline upwards of 50% (Beenakker et al., 2010) with most of that decline occurring after ~65 to 70 years of age (Sternäng et al., 2015). Sensation of the fingers is also altered with age, including increased tactile thresholds (Bruce, 1980; Bowden and McNulty, 2013; Decorps et al., 2014; Thornbury and Mistretta, 1981) and poorer ability to discriminate between different surfaces (Bowden and McNulty, 2013; McIntyre et al., 2021; Skedung et al., 2018) and patterns (Logue et al., 2022; Master et al., 2010).

Apart from strength and sensation, hand function is heavily dependent on the ability to manipulate objects. Hand dexterity, a broad component of hand function which also encompasses object manipulation, has recently been defined by Yong et al. (2020) as “the coordination of voluntary movement to accomplish an actual or simulated functional goal/task accurately, quickly, resourcefully and adapting to environment or change”. Similarly to strength and sensation, age-related declines in hand dexterity (Carmeli et al., 2003; Ranganathan et al., 2001a), proprioception (Goble et al., 2009; Hughes et al., 2015), and upper extremity movement coordination (Brown, 1996; Heintz Walters et al., 2021; Wittenberg et al., 2022) have also been well documented. For example, dexterity pegboard assessments have demonstrated age-related declines ranging from 20 to 50% between young and older adults (Bowden and McNulty, 2013; Kobayashi-Cuya et al., 2018; Marmon et al., 2011; Ranganathan et al., 2001a). We, along with others, have also demonstrated greater errors in proprioceptive matching tasks among older adults (Adamo et al., 2007, 2009; Goble et al., 2012; Landelle et al., 2018; Schaap et al., 2015; Wright et al., 2011), particularly during movements that involve interhemispheric transfer or when attentional demands are added (Adamo et al., 2009; Goble et al., 2012; Herter et al., 2014). Submaximal hand force control, sometimes referred to as fine force control, including both the generation and maintenance of force, is also poorer among older adults compared to young. For example, older adults generate force less smoothly (Logue et al., 2022) and less accurately (Shim et al., 2004) than young adults and have poorer hand steadiness (Logue et al., 2022; Marmon et al., 2011; Martin et al., 2015; Ranganathan et al., 2001a; Strote et al., 2020; Tracy et al., 2005), particularly at very low force levels (e.g., 5–10% maximum voluntary contraction) (Logue et al., 2022; Tracy et al., 2005). Further, aging is associated with a reduction in the rate at which grip force is developed (Bellumori et al., 2013), particularly in females (Corrêa et al., 2020) which has consequences for rapid adjustments in the face of unexpected perturbations (Bellumori et al., 2013). Such declines in force development are also exacerbated by increased motor unit discharge variability (Enoka et al., 2003; Tracy et al., 2005) and increased antagonist coactivation (Latash, 2018; Spiegel et al., 1996) with age. Combined, older adults may have greater difficulty handling objects that require precise control and may either generate excess force to prevent slipping (Olafsdottir et al., 2007) or, in the case of fragile objects, too little force to prevent crushing, thereby increasing the risk of slipping (Gorniak et al., 2011; Hibino and Gorniak, 2020).

As mentioned previously, the aging hand receives far less attention than other aspects of health, for example balance and posture. However, most activities of daily living require adequate use of the hands, thus hand impairments can greatly impact one’s ability to live independently. For example, basic care tasks such as hearing aid handling (Singh et al., 2013), eye drop installation (Weber et al., 2025), opening medication bottles (Angel et al., 2022), dressing, and eating can be impacted by the loss of hand function and control. Several studies have demonstrated that hand function, specifically dexterity as opposed to strength, is a strong indicator of disability status (de Paula et al., 2016; Jette et al., 1990), frailty (Beier et al., 2022), and dependency (Falconer et al., 1991; Ostwald et al., 1989) and better predicts dependence than lower limb function (Jette et al., 1990; Ostwald et al., 1989). Further, poorer dexterity is associated with increased risk of cognitive decline (Carment et al., 2018; Hesseberg et al., 2021; Kobayashi-Cuya et al., 2018), dementia (Menengic et al., 2023; Darweesh et al., 2017), and brain atrophy (Dougherty et al., 2024). While dexterity may be a better indicator of functional status than maximum strength, there is evidence to suggest that weaker grip strength is predictive of increased DNA methylation, a biomarker of accelerated aging (Peterson et al., 2023). Age-related hand impairments are also quite common, with population data revealing that over 30% of U. S. older adults report limitations with hand-related activities (Logue Cook et al., 2022) and that age-related hand limitations are more prevalent among minority groups, specifically those who identify as Mexican American (Logue Cook et al., 2024a).

## 3 Declining hand function in older adults – the need for better clinical assessment tools

While balance assessments such as the Romberg or the unipedal stance test (Nnodim and Yung, 2015) are commonly part of routine clinical visits for older but otherwise healthy adults, clinical evaluation of hand function is often not addressed or involves grasping the examiner’s hand as hard as possible. This approach can reveal overt reductions in hand strength but does not have the sensitivity to detect subtle deteriorations in fine force control or impaired dexterity, both of which are essential for daily activities. Referrals to specialists primarily occur only when there is a suspected injury or condition that impairs hand function such as the presence of pain and/or reduced grip strength (Leow et al., 2019). Depending on the diagnosis, nonsurgical treatment typically includes exercises targeting muscle strength, range of motion, and dexterity (O'Connell et al., 2025).

As described earlier, the control of low hand forces typically required for activities of daily living is compromised in older adults and may be independent of any changes in age-normative maximum strength (Carment et al., 2018; Logue et al., 2022). Similarly, age-related declines in tactile acuity may not be detected using standard clinical measures. For example, age-related declines in the ability to recognize spatial tactile patterns may occur in the absence of any changes based on standard clinical assessments such as monofilament testing (Logue et al., 2022). Further, health care providers typically do not ask about hand function and older adults often assume that declines in hand function are “just part of aging” (Logue Cook et al., 2026). As a result, they do not report subtle changes in hand use and may alter or limit daily activities to accommodate such declines.

Thus, there is a clear need to develop objective and comprehensive assessments that more closely reflect daily hand tasks. For example, Lawrence et al. (2015) identified three domains of hand function: sensorimotor processing, upper limb coordination, and strength that need to be considered when determining age-related changes in functional hand use. This recommendation is supported by a growing body of evidence that maximum grip strength may not be predictive of object manipulation skills needed for activities of daily living (Dayanidhi and Valero-Cuevas, 2014; Kobayashi-Cuya et al., 2018; Logue et al., 2022; Logue Cook et al., 2022). As such, multifactorial hand assessments are needed which measure not only strength, but other aspects of daily hand use requiring dexterity and tactile acuity and which are easily accessible in the clinical setting.

## 4 Improving hand function in older adults – effects of hand training

In contrast to a variety of exercise-based interventions targeting muscle strength and balance control in an effort to reduce fall risk (Pillay et al., 2024; Cabrolier-Molina et al., 2025), there are far fewer studies focused on maintaining or improving hand function in healthy older adults. While whole body training interventions such as Tai Chi (Huang et al., 2022) and dance (Lazo Green et al., 2024) have been used primarily to improve balance (Huang et al., 2022), there is evidence that such interventions can also improve tactile perception (Kerr et al., 2008) and hand dexterity (Kattenstroth et al., 2013). It has been suggested that such non-hand specific exercise may contribute to improved hand sensorimotor control due to neurotrophic-mediated enhancement of synaptic efficacy as well as enhanced cognitive function (Kattenstroth et al., 2010, 2013).

Ranganathan et al. (2001b) demonstrated that practicing rotation of two metal balls in the palm of the hand over an 8-week period significantly improved submaximal force control and dexterity. Several weeks of finger strength training led to improved maximum strength and force control although improvements in dexterity were only seen for pegboard tests and not for more complex manipulation tasks (Olafsdottir et al., 2008) Strength or dexterity training has led to improvements over shorter periods of 1–2 weeks (Francis et al., 2012; Marmon et al., 2011; Pereira et al., 2011) which presumably reflect rapid neuromuscular adaptations leading to improved control of motor unit activation patterns (Kornatz et al., 2005). Bimanual hand training has also led to improved dexterity in older adults, thought to be the result of trained-induced reduction in ipsilateral motor-cortical activity (Naito et al., 2021). More recently, Park et al. (2025) demonstrated the value of unilateral dominant hand strength training on dexterity in both hands, suggestive of cross educational benefits for maintenance of hand function in older adults.

Despite the importance of somatosensory feedback for precise motor tasks and the known age-related declines in proprioception, few hand-specific studies have examined whether movement-based interventions enhance the perception and utilization of such feedback. Recently, we demonstrated that a six-week, home-based training program focusing on fine force control, object manipulation, and somatosensory training led to significant improvements in not only dexterity but also tactile acuity, and submaximal force perception (Logue Cook et al., 2024b). Performance on the Trail Making Test Part B - a measure of cognitive flexibility - also improved, confirming previous work demonstrating the beneficial effects of ha d training on cognition (Seol et al., 2023). It is important to note that delivery of hand training programs does not need to be limited to clinical or research settings. For example, we recently demonstrated that a community-based, intergenerational hand training program for homebound older adults demonstrated improvements in pinch strength (Logue Cook et al., 2023). Taken together, hand specific training interventions to maintain or improve function in older but otherwise healthy older adults should include a variety of components that target hand dexterity, fine force control, bimanual coordination, range of motion, as well as overall strength. Simple exercises can be easily designed incorporating everyday objects or games (Logue Cook et al., 2023, 2024b), although future studies are needed to determine optimal dose and retention of performance gains.

## 5 Other factors affecting hand function in older adults

**Figure 2:**
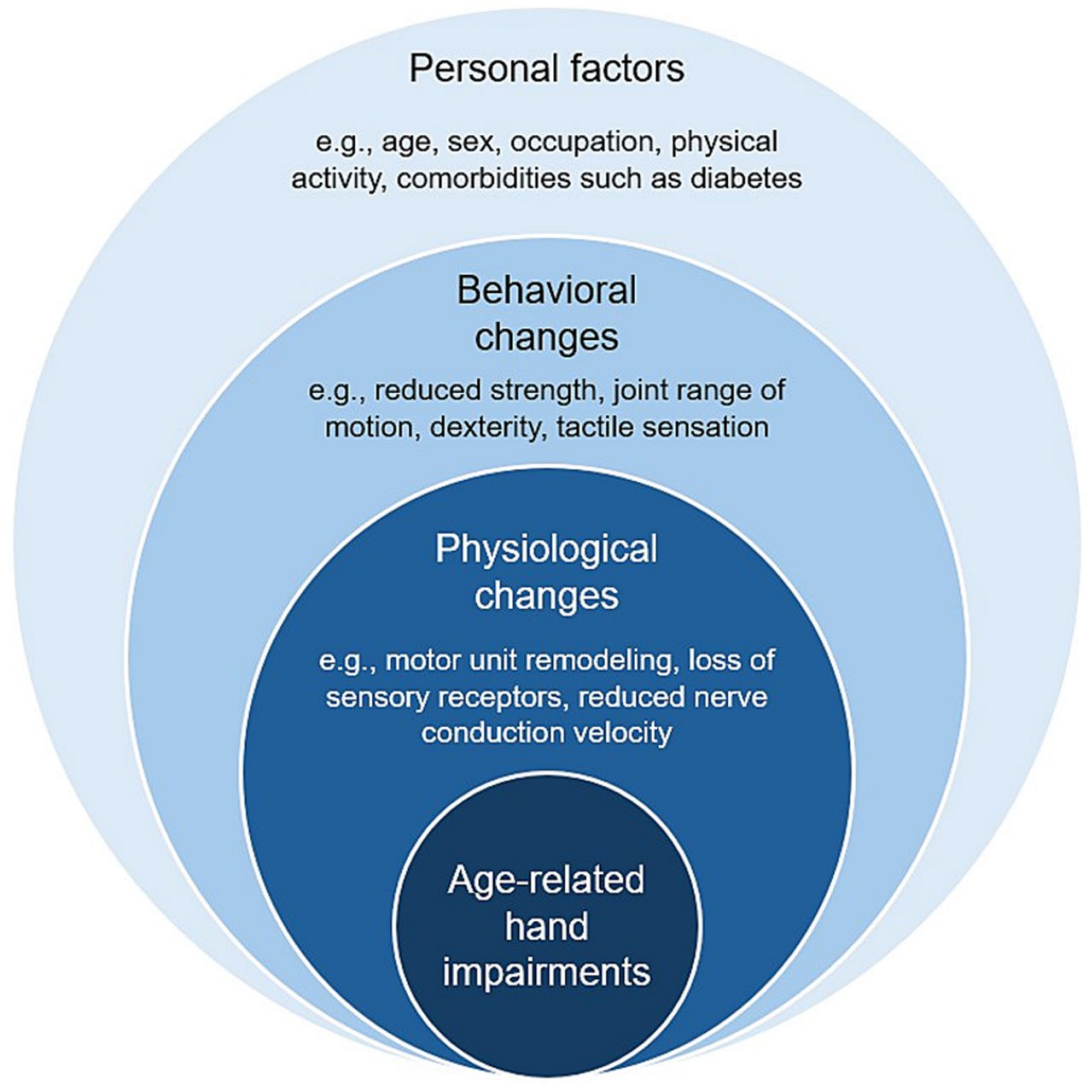
Factors affecting the development of age-related hand impairments.

Apart from age-related declines in various body systems affecting hand use, other factors can impact hand sensorimotor function including sex differences, past occupational history, and comorbid conditions (Figure 2). It is well established that maximum grip strength is greater in males than females (Song et al., 2025; Tomkinson et al., 2025), with age-related declines occurring at a faster rate in older women (Huebner et al., 2022). When considering hand dexterity, however, sex differences vary depending upon the task. While a female advantage exists for visually guided fine force control tasks involving object manipulation (Desrosiers et al., 1995; Lezak et al., 2012; Ranganathan et al., 2001a; Vasylenko et al., 2018), greater variability in maintaining low force levels have been observed in women (Ranganathan et al., 2001a). Several factors may contribute to such sex-related differences in older adults including alterations in motor unit recruitment patterns at low force levels (Lulic-Kuryllo and Inglis, 2022) as well as differences in lifestyle activities (Merritt and Fisher, 2003), functional brain connectivity (Rogojin et al., 2023), and/or movement strategies (Rohr, 2006).

Sedentary lifestyles also contribute to an accelerated loss of muscle strength with age (Steffl et al., 2017), including greater reductions in grip strength in post-menopausal (Alzuwaydi et al., 2025) and older women (Lee et al., 2020). We have found that the ability to reproduce wrist joint angles based solely on position sense was worse in older sedentary compared to active older adults (Adamo et al., 2009). Such declines were particularly noticeable in tasks requiring contralateral matching of a memory-based reference joint position requiring interhemispheric transfer of target position. Similarly, low physical activity levels were associated with poorer detection of wrist movement (kinesthesia) (Wright et al., 2011). These findings may reflect the established benefits of physical activity on cognitive and perceptual-motor skills (Erickson et al., 2019), critical for non-visual monitoring of static and dynamic hand position.

Longterm occupational hand use requiring expert object manipulation skills and/or tactile perception has been associated with enhanced tactile pattern accuracy in late middle-aged individuals (Reuter et al., 2014). Depending upon the intensity and type of occupational hand use, however, older adults are at higher risk for developing musculoskeletal conditions such osteoarthritis and carpal tunnel syndrome that negatively impact daily living (Minetto et al., 2020). For example, we recently provided evidence that older blue-collar workers were more likely to report hand impairments compared to those who had worked in white collar positions, possibly due to subclinical overuse disorders and occupation-related disparities in health care access (Levinson et al., 2025). However, to what extent past occupations may contribute to racial/ethnic differences in self-reported impairments in hand-specific activities of daily living in older adults remains to be determined (Logue Cook et al., 2024a). There is also evidence of the relationship between education and socioeconomic status on functional performance related to daily activities where hand use is critical (Homann et al., 2003; Carney and Benzeval, 2018). Lastly, the increased prevalence of hand injuries (Fulchignoni et al., 2023) as well as the development of comorbid conditions such as joint deformities (Dziedzic et al., 2007) and peripheral neuropathies (Niu et al., 2023) can further limit functional hand use in older adults. Future studies should also consider the impact of pain and additional age-related conditions such as vision loss and their interactions with hand function with age.

## 6 Conclusion

The goal of this review is to increase awareness of the importance of maintaining or improving hand function in our increasingly aging segment of society – it’s not just about muscle strength. The human hand is a remarkable sensorimotor organ that provides us with the ability to perform most if not all activities of daily living. It can also serve as a substitute for verbal communication and is the substrate for an untold number of creative and artistic endeavors. Yet, in the absence of comorbid conditions, attention to hand function in late adulthood is primarily focused on maximum hand strength given its ability to predict long term health outcomes in older adults. For daily activities, however, efficient hand function is dependent upon dexterous manipulation skills and the precise control of low forces. Since age-related declines in hand force production, coordination, and utilization of somatosensory feedback are well established, the purpose of this review was to refocus attention on changes in hand dexterity and force modulation that are critical for functional independence.

There is a growing body of research supporting the value of targeted hand training to maintain and/or improve hand dexterity and fine force control in healthy older adults. From a health care perspective, however, older adults and care providers have little access to such information and thus, hand function is not routinely assessed. This is due, in part, to a lack of objective hand assessment tools that can be easily used in health care settings. Future research should also include the development of telehealth applications that increase access to stand alone hand training programs or supplement existing balance training protocols.
